# Trends and future projections of liver cancer incidence in Hong Kong: a population-based study

**DOI:** 10.1186/s13690-023-01191-3

**Published:** 2023-10-03

**Authors:** Tianyou Ma, Xiaohui Wei, Xiaoming Wu, Jianqiang Du

**Affiliations:** 1 School of Public Health, Laboratory for Disease Prevention and Control and Health Promotion of Shaanxi Province, Xi’an Jiaotong University Health Science Center, Xi’an, Shaanxi China; 2 Third Department of Medical Oncology, Shaanxi Provincial Cancer Hospital Affiliated to Medical College of Xi’an Jiaotong University, Xi’an, Shaanxi China; 3 The Key Laboratory of Biomedical Information Engineering of Ministry of Education, School of Life Science and Technology, Xi’an Jiaotong University, No.28, Xianning West Road, Xi’an, 710049 Shaanxi China

**Keywords:** Liver cancer, incidence, Period analysis, Cohort effect, Demographic factors

## Abstract

**Background:**

Liver cancer remains a significant burden in Hong Kong. We sought to examine trends in liver cancer incidence using 30 years of cancer registry data in Hong Kong. Additionally, we aimed to assess the impact of age, period and birth cohort on liver cancer incidence, provided projections for liver cancer incidence until 2030, and examined the drivers of liver cancer incidence.

**Methods:**

Data on liver cancer incidence were collected from the Hong Kong Cancer Registry (HKCaR). We assessed age, period, and birth cohort effects using age-period-cohort (APC) models. We employed Bayesian APC analysis with integrated nested Laplace approximations to project the future burden of liver cancer in Hong Kong. Furthermore, we attributed the changes in new liver cancer cases to population growth, population ageing, and epidemiological changes.

**Results:**

The study included a total of 51,333 individuals, of whom 39,287 (76.53%) were male. From 1991 to 2020, the age-standardized liver cancer incidence rate in Hong Kong continued declining, while the number of new cases increased significantly, especially among males. The net drift, representing the overall annual percentage change of the age-adjusted rate, was − 3.06% (95% confidence interval [CI]: -3.31% to -2.80%) for males and − 3.85% (95% CI: -4.61% to -3.09%) for females. Local drift, which estimates the annual percentage change over time specific to age group, decreased in all age groups for both sexes, with a more pronounced decrease in younger age groups. The period and cohort risk of developing liver cancer also showed decreasing trends for both sexes. The study projected a decline in liver cancer cases for males but an increase for females in Hong Kong, with an estimated 1,083 cases in males and 710 cases in females by 2030. Demographic decomposition analysis revealed that while population growth and ageing were the main drivers of increased liver cancer cases, epidemiologic shifts mostly offset these factors.

**Conclusion:**

The period and cohort risk of developing liver cancer in Hong Kong declined due to epidemiological changes. Although the age-standardized incidence rates of liver cancer have also declined, demographic and epidemiological factors have led to lower case expectations in males but a likely increase in females. Further research and epidemiological assessment of the disease are needed.

**Supplementary Information:**

The online version contains supplementary material available at 10.1186/s13690-023-01191-3.

**Table:** 

**Text box 1. Contributions to the literature**
• This research paper presents a comprehensive analysis of liver cancer incidence trends in Hong Kong over a 30-year period, highlighting the changing patterns and drivers of liver cancer in the population.
• By employing advanced statistical models, the study reveals declining period and cohort risks of developing liver cancer, indicating positive epidemiological changes in the region.
• The paper provides projections for liver cancer incidence until 2030, showing a projected decline in cases for males but an expected increase for females, emphasizing the need for targeted interventions and further epidemiological research in the context of gender-specific disparities.

## Introduction

Liver cancer is the sixth most common neoplasm and the third leading cause of cancer mortality globally in 2020 [1, 2], with the highest incidence in Eastern Asia. According to the most recent statistics in 2020 of the Hong Kong Cancer Registry (HKCaR) [3], liver cancer is the fifth most often diagnosed cancer and the third most common cause of cancer deaths in Hong Kong. Over the last three decades, the incidence of liver cancer in Hong Kong has shown a remarkable decline, aligning with the overall trend observed in Eastern Asia [1, 4].

Hepatocellular carcinoma (HCC) is the predominant histologic type of liver cancer in Hong Kong [5, 6]. The major causes of HCC are chronic infection with hepatitis B virus (HBV) or hepatitis C virus (HCV), exposure to aflatoxin-contaminated food, excessive alcohol use, obesity, type 2 diabetes, and smoking [1, 2]. However, the incidence of HCC in Hong Kong may not be related to either HCV infection or aflatoxin contamination, as the prevalence of HCV in Hong Kong is low, at 0.3% [7], and aflatoxin contamination is rare [8]. Over the past three decades, the incidence of liver cancer in Hong Kong has declined, mainly due to the decreasing prevalence of HBV in the population [9–11]. Furthermore, as the prevalence of HBV decreases, other risk factors for liver cancer, such as obesity and type 2 diabetes, may become more prevalent and shift the pattern of liver cancer in Hong Kong.

The risk of developing liver cancer varies between birth cohorts due to differences in the prevalence of HBV and HCV infection and other risk factors over time [1, 2, 9–12]. Age-period-cohort (APC) modelling can separate and analyze the effects of temporal changes and birth cohort variation on the incidence of liver cancer [13, 14]. Knowledge of the incidence trends of liver cancer in Hong Kong can improve disease prevention efforts. In this study, we analyzed the incidence trend of liver cancer in Hong Kong using high-quality population-based cancer registry data. We also examined how age, calendar period, and birth cohort interact and are related to liver cancer incidence in Hong Kong. Furthermore, we projected the future incidence of liver cancer in Hong Kong up to 2030 and decomposed changes in new liver cancer cases into demographic and epidemiological factors.

## Materials and methods

### Data source

Data on liver cancer incidence in Hong Kong between 1991 and 2020 were obtained from the Hong Kong Cancer Registry (HKCaR) [3], a population-based cancer registry encompassing the entire population of Hong Kong. Liver cancer cases were classified using the International Classification of Diseases, 9th code 155 and 10th code C22. For the analysis, individuals under 20 were excluded due to the rarity of liver cancer in this age group. Population estimates by year and age group were obtained from the 2019 Revision of the United Nations (UN) World Population Prospects (based on the UN medium-fertility variant) [15], which is the 26th edition of the UN’s official population estimates covering 235 countries. The UN World Population Prospects provide historical demographic trends and population projections until 2100. Standardized incidence rates were calculated according to the World Health Organization’s World standard population. Institutional review board approval was not required as the data used in this study were publicly available and did not involve any identifiable information.

### Age-period-cohort analysis

The study employed the age-period-cohort (APC) approach to evaluate the effects of age, period, and birth cohort on liver cancer incidence. The incidence and population data were arranged into 14 five-year age groups (ranging from 20 to 24 years to 85 + years) and six five-year calendar periods (from 1991 to 1995 to 2016–2020), which allowed for the definition of 17 birth cohorts (from 1904 to 1908 to 1994–1998). Within the APC models, net drift and local drift were key parameters [16, 17]. Net drift represents the estimated annual percentage change of age-adjusted rates over time, while local drift refers to the estimated annual percentage change specific to each age group over time. Period effects capture changes that co-occur across all age groups, typically arising from alterations in social or economic environments. Cohort effects pertain to changes observed within groups of individuals sharing the same birth year. Period and cohort effects are represented as rate ratios relative to the central calendar period and cohort, which were selected as the reference. The APC analysis was conducted using a web-based tool provided by the National Cancer Institute [16]. The significance of the estimates was determined using the Wald chi-square statistic. All statistical tests were two-sided, and a p-value of less than 0.05 was considered statistically significant.

### Projection

The Bayesian APC framework is a statistical approach incorporating prior knowledge into the model to estimate projection uncertainty [18]. This method provides a more reliable projection of future trends. The integrated nested Laplace approximation (INLA) is a computational tool that fits complex statistical models faster and more accurately than traditional methods [19]. To project the incidence and new cases of liver cancer in Hong Kong from 2021 to 2030, we utilized the Bayesian APC framework with INLA in our projection algorithm. The R package BAPC was used to fit our model and perform the projections [18, 20, 21]. The population estimates used in our projections were derived from the United Nations Population Division’s World Population Prospects.

### Decomposition

We partitioned the changes in the number of new liver cancer cases in Hong Kong from 1991 to subsequent years between 1992 and 2030 into three factors: population ageing, population growth, and age-specific incidence rate (i.e. epidemiological changes). Epidemiological changes encompass shifts in disease risks and advances in diagnostic practices. We utilized a validated algorithm to perform the decomposition, which was robust to the decomposition order and the reference year selection [22, 23]. Further information about the decomposition method can be found in Appendices S1 and S2 of the supplementary materials. The data were handled and analyzed using R version 3.6.3.

## Results

### Trends in the incidence of liver cancer

**Fig. 1:**
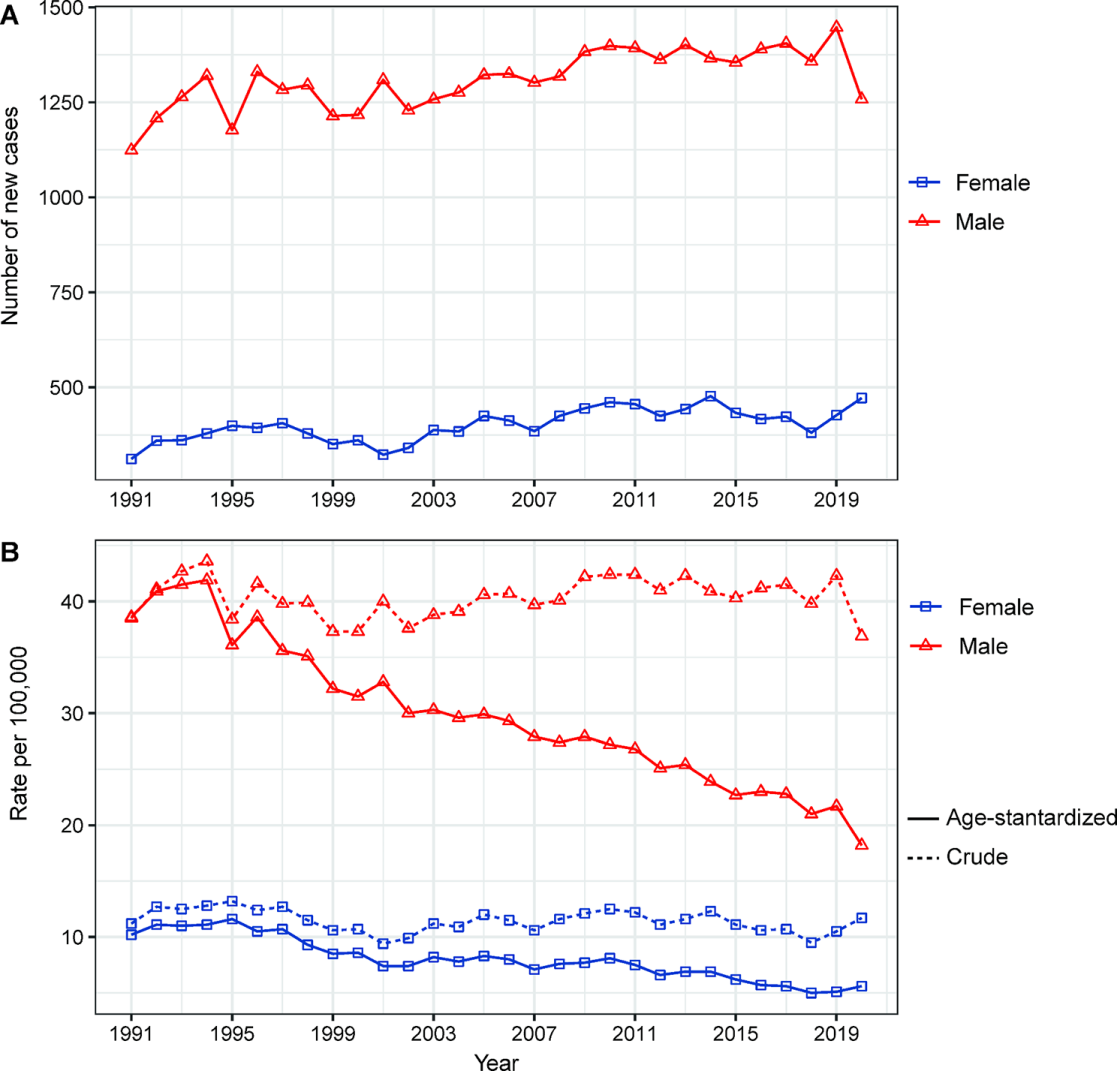
Changes in the incidence rate and number of cases of liver cancer in Hong Kong from 1991 to 2020. **A**, Number of cases. **B**, Age-standardized incidence rate and crude incidence rate

Between 1991 and 2020, a total of 51,333 liver cancer cases were diagnosed in Hong Kong, with 39,287 cases (76.53%) occurring in males and 12,046 (23.47%) cases in females. The number of liver cancer cases increased over time for both males and females, rising from 1,124 to 312 cases in 1991 to 1,258 and 472 cases in 2020, respectively (Fig. 1A). While the crude incidence rate of liver cancer remained relatively stable for both sexes, the age-standardized incidence rate significantly declined, particularly among males. Specifically, the age-standardized incidence rate of liver cancer decreased 38.6 to 18.2 cases per 100,000 population for males and from 10.2 to 5.6 cases per 100,000 population for females, between 1991 and 2020 (Fig. 1B).

### APC modelling

**Fig. 2:**
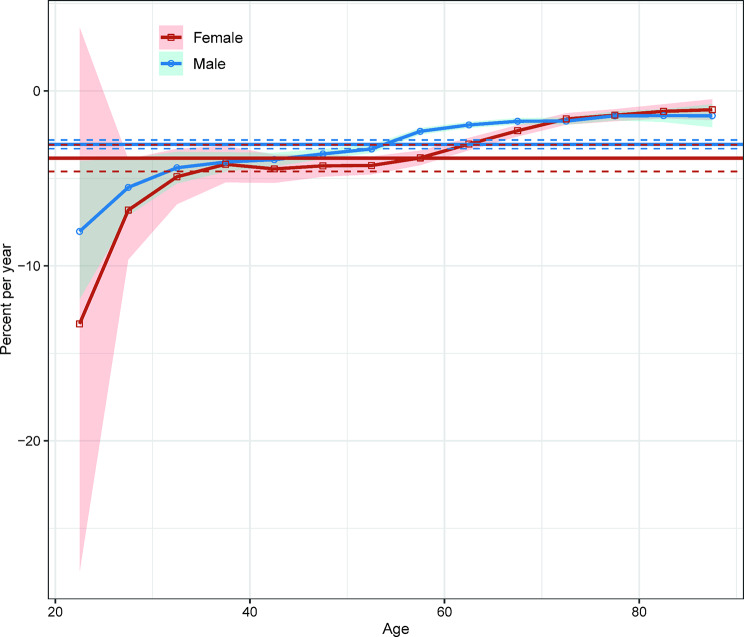
Local drifts with net drift values for liver cancer incidence in Hong Kong from 1991 to 2020. The horizontal solid line and corresponding dashed lines represent the net drift and its 95% confidence intervals, while the curve and corresponding shaded area represent the local drift and its corresponding 95% confidence intervals

The net drift showed a statistically significant downward trend in the incidence of liver cancer, with a decrease of -3.06% (95% confidence interval [CI]: -3.31% to -2.80%) per year in males and − 3.85% (95% CI: -4.61% to -3.09%) per year in females over the entire period (Fig. 2). However, the local drifts, which capture age-specific changes in incidence trends, showed considerable heterogeneity. Decreases were observed across all age groups for both sexes, with more pronounced declines in the younger age groups and smaller in older age groups (Fig. 2).

**Fig. 3:**
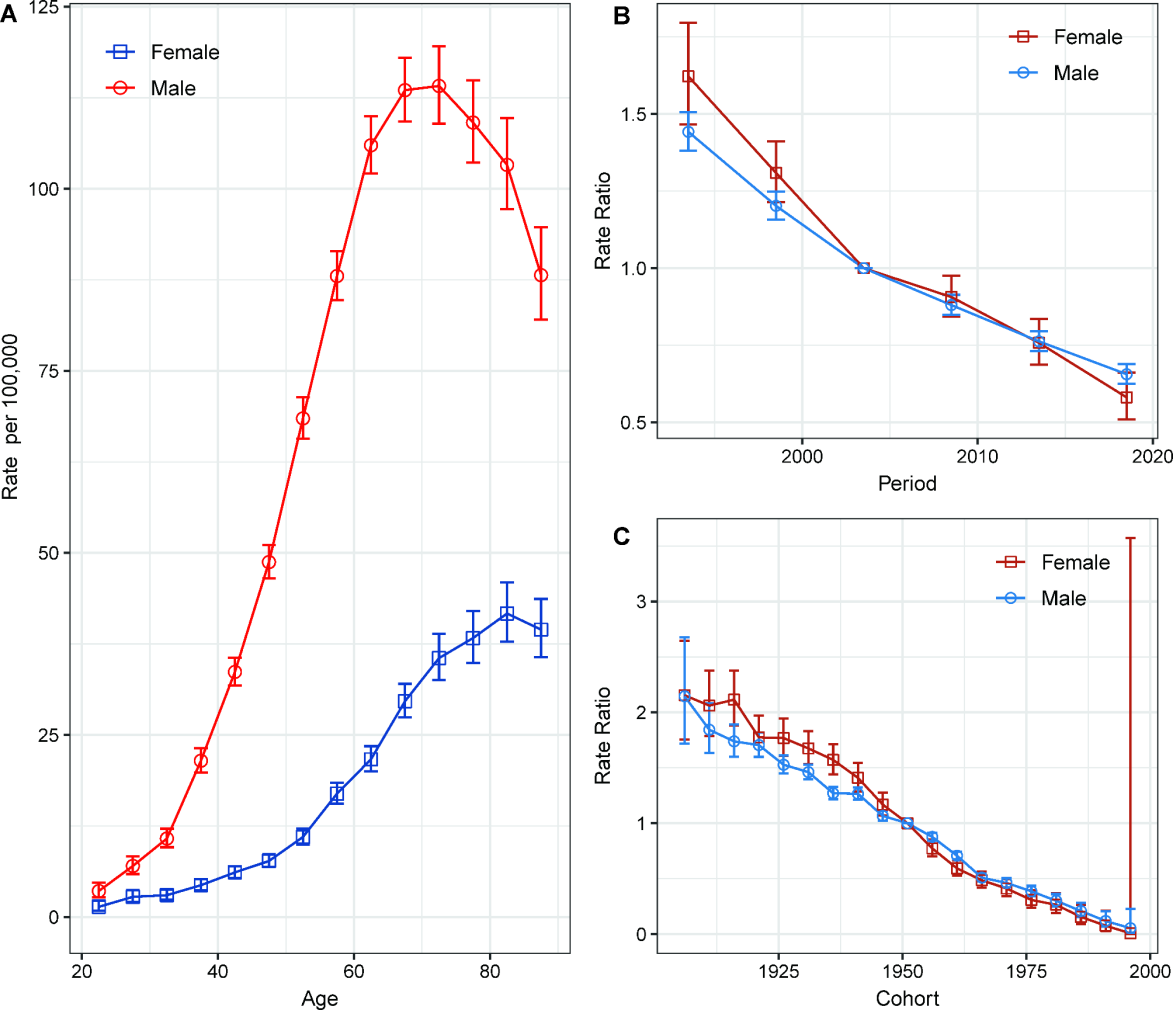
Age, period, and birth cohort effects with the corresponding 95% confidence intervals on liver cancer incidence rates in Hong Kong from 1991 to 2020. (A) Longitudinal curves of fitted age-specific rates in reference cohort adjusted for period effects; (B) Rate ratios in each period relative to the reference period; (C) Rate ratios in each cohort relative to reference cohort

Furthermore, after adjusting for period deviations, we found that the incidence of liver cancer in males increased with age, peaked around the age of 70, and then declined. On the other hand, in females, the incidence rates increased with age (Fig. 3A). We employed a second-order polynomial regression model, which provided a good fit for describing the relationship between liver cancer incidence rate and age. The model had an R-squared value of 0.998 for males and 0.986 for females, indicating that it explained 99.8% of the variation in incidence rates for males and 98.6% for females.

The period rate ratios for both males and females exhibited a monotonic decline, with females experiencing a more pronounced decrease, indicating a more marked period effect (Fig. 3B). In addition, the cohort rate ratios for both sexes decreased monotonically, suggesting the presence of cohort effects (Fig. 3C). Wald tests confirmed that the main estimable functions were statistically significant for both sexes (p < 0.05) (Table S1).

### Projection

**Fig. 4:**
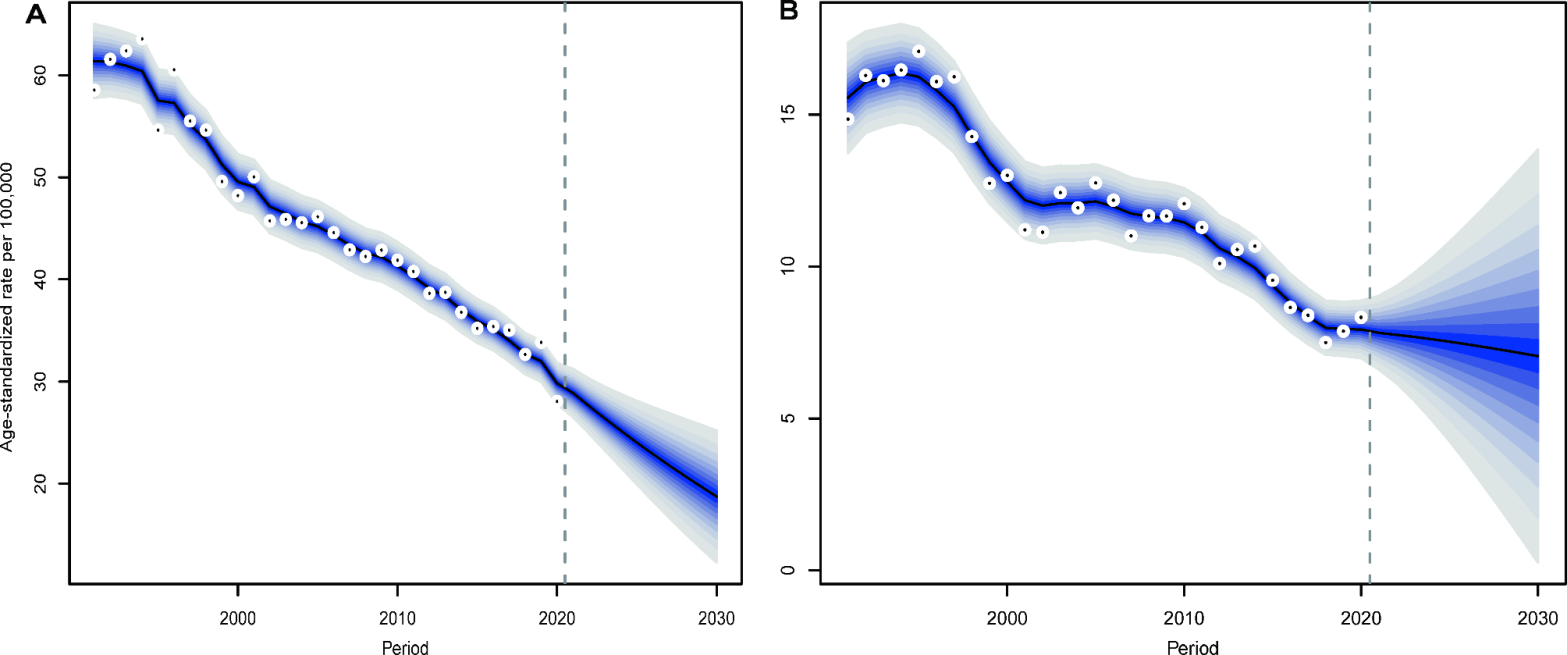
Trends and projected incidence rates for liver cancer in Hong Kong. A. For males; **B**. For females. Data to the right of the dashed line are projected data. Each lighter shade of blue represents an additional 10% CI.

Our projections indicate that the number of liver cancer cases in Hong Kong will follow different trends for males and females. The number of new cases in males is projected to decrease from 1,258 to 2020 to 1,083 in 2030, while in females, it is expected to increase from 472 to 710 during the same period (Tables S2, S3). The highest increase in incident cases is predicted among the elderly population aged over 70 years. Furthermore, both sexes are expected to experience a continued decline in the age-standardized incidence rate (Fig. 4).

### Decomposition

**Fig. 5:**
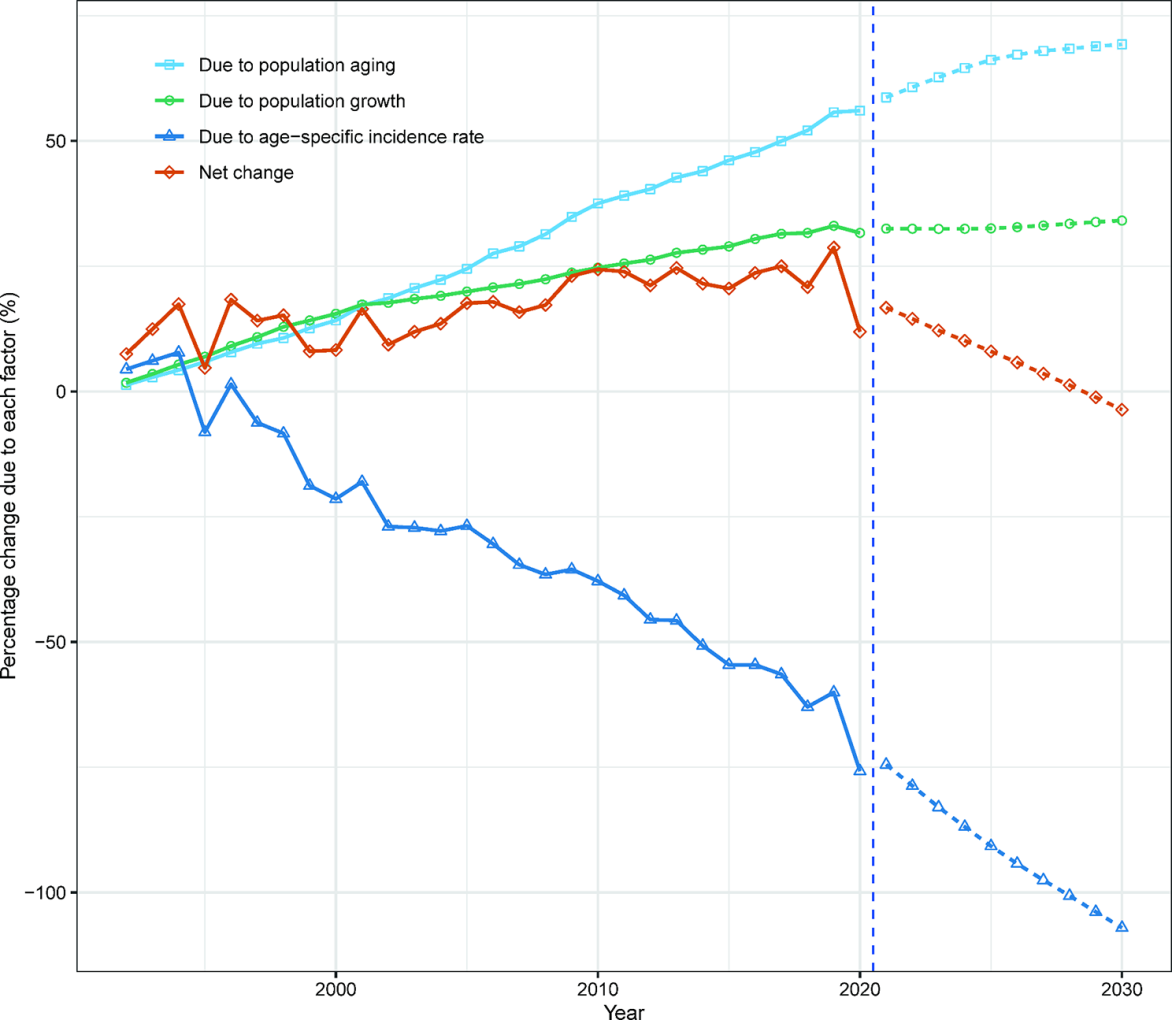
Contribution of changes in population ageing, population growth, and age-specific incidence rate to changes in new liver cases from 1992 to 2030 for Hong Kong males, using 1991 as the reference year. Data to the right of the blue dashed line represent the decomposition based on the projected data

**Fig. 6:**
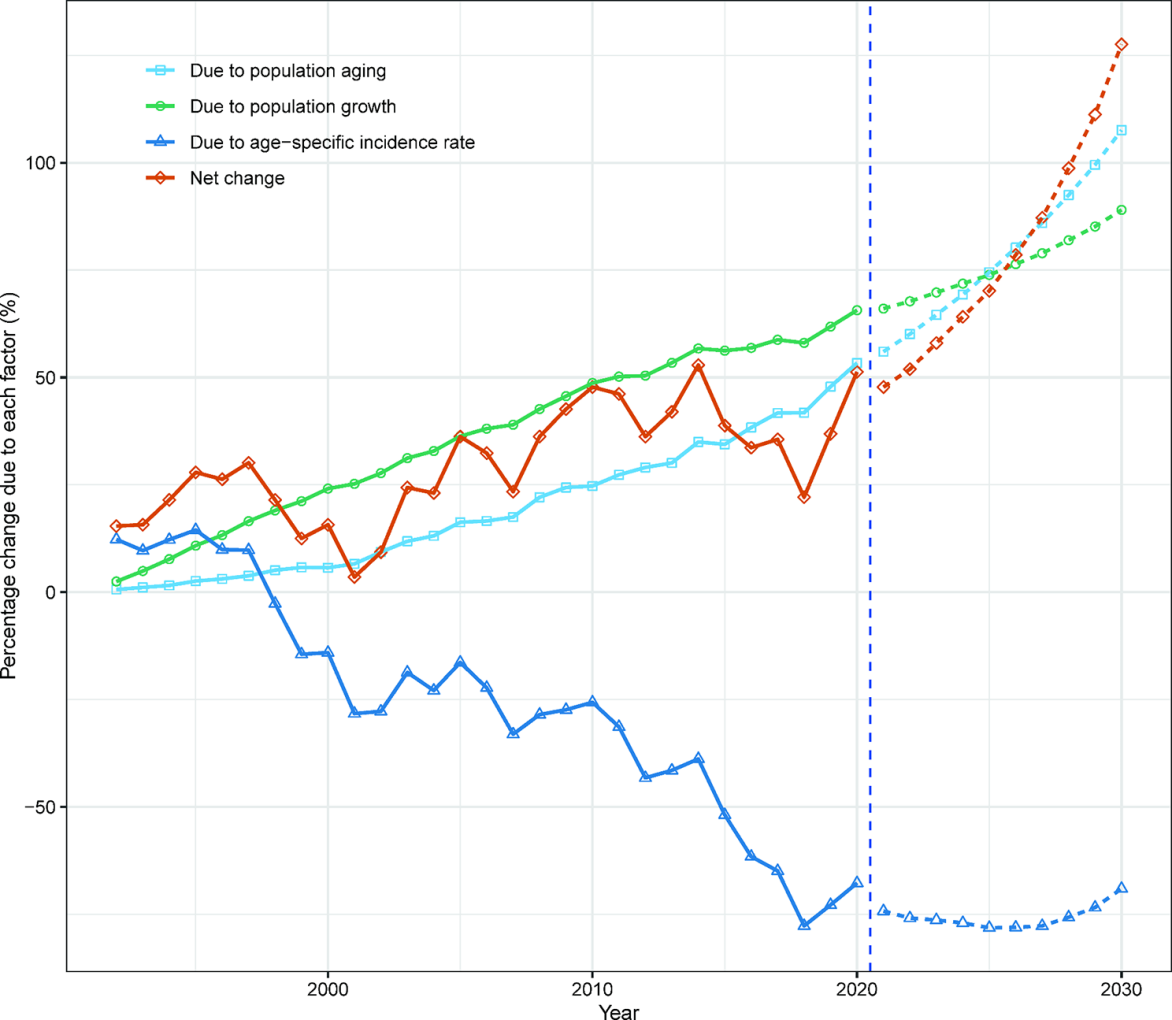
Contribution of changes in population ageing, population growth, and age-specific incidence rate to changes in new liver cases from 1992 to 2030 for Hong Kong females, using 1991 as the reference year. Data to the right of the blue dashed line represent the decomposition based on the projected data

Compared to 1991, the number of new liver cancer cases in Hong Kong increased by 11.9% (134 cases) for males and 51.3% (160 cases) for females in 2020. This increase can be primarily attributed to the ageing population, accounting for 56.1% (630 cases) for males and 53.4% (167 cases) for females, population growth accounting for 31.6% (356 cases) for males and 65.7% (205 cases) for females, and epidemiological changes accounting for − 75.8% (-852 cases) for males and − 67.7% (-211 cases) for females (Figs. 5 and 6; Tables S4, S5).

According to our projections, the number of new liver cancer cases in males in males is expected to decrease from 1,258 to 2020 to 1,083 in 2030. Conversely, we anticipate an increase from 472 to 710 for females over the same period. While the age-standardized incidence rate of liver cancer in Hong Kong may continue to decline, the decline for females is smaller than that for males (Fig. 4). Our decomposition analysis shows that the decrease in male liver cancer cases due to demographic factors will be fully offset by epidemiological factors, resulting in a net reduction. However, for females in Hong Kong, epidemiological factors will not be able to completely offset the impact of demographic factors, leading to a net increase in the number of liver cancer cases among females (Figs. 5 and 6; Tables S4, S5).

## Discussion

In this study, we employed APC analysis to determine the causes of the incidence trend of liver cancer in Hong Kong. We observed a significant decline in the age-standardized incidence rate of liver cancer in both sexes. Although demographic changes increased the number of liver cancer cases, they were primarily offset by epidemiological changes. We identified a decreasing trend in the period and cohort risk of developing liver cancer for both sexes. Our research findings demonstrated that demographic factors account for the majority of new liver cancer cases in Hong Kong, and the diminishing cohort effects suggest a transition in the risk factors for liver cancer in the population. Continued epidemiological surveillance and preventative approaches are essential for addressing this disease.

The declining trend in the period and cohort risk of developing liver cancer in Hong Kong is encouraging. It suggests that the efforts to prevent and control hepatitis B have been effective. In Hong Kong, the seroprevalence of hepatitis B surface antigen (HBsAg) has declined in several populations with no obvious HBV risk, such as new blood donors and pregnant women [24]. Hong Kong has transitioned from a high-intermediate to an intermediate-low hepatitis B endemicity over the last few decades. In addition, antiviral treatment for hepatitis B carriers in the late 1990s has led to a sustained decline in the period risk of developing liver cancer [25, 26]. According to a series of vaccination coverage surveys [27], the coverage of the third dose of the hepatitis B vaccine has consistently been around 99% or higher among children aged 3–5, implying that the cohort risk of developing liver cancer will continue to wane for birth cohort after 1988. Adults in their 30s who did not receive protection from the universal neonatal hepatitis B vaccination program, which began in 1988, bear the burden of HBV infection. Recent research indicated that the overall HBsAg seroprevalence is as high as 7.2% [7], implying that over 500,000 people have chronic hepatitis B in Hong Kong. To further reduce the burden of liver cancer in Hong Kong, it is essential to improve prevention measures against mother-to-child transmission and provide access to appropriate medical management for those who have chronic hepatitis B.

Another significant contribution of this study is predicting the trend of liver cancer incidence in Hong Kong. Our analysis indicates a significant downward trend in the age-standardized incidence of liver cancer, but the number of cases remains high. The inconsistency is mainly due to demographic changes in Hong Kong, namely, ageing. In addition to viral infections, other factors such as excessive alcohol consumption, overweight, type 2 diabetes, and smoking may increase, particularly in males [23, 28]. Demographic changes, such as population ageing and growth, may exacerbate the role of these risk factors in liver cancer development. In individuals without hepatitis virus infection, the abovementioned risk plays a more significant role in developing liver cancer. According to the Hong Kong Department of Health [27], 8.8% of people aged 15 and above drink alcohol regularly, with the highest rate of 12% observed among those aged 55–64. Additionally, 29.9% of the population is obese, and 20.1% are overweight. The prevalence of diabetes is 8.4%, and the prevalence of smoking remains above 10.5%. As the population ages, the increasing incidence of diabetes and obesity [23, 28] will continue to contribute to the development of liver cancer, and the proportion of liver cancers caused by metabolic disorders will increase. However, it should be noted that our dataset lacks specific data on risk factors.

In Hong Kong, liver cancer incidence is significantly higher in males than females, with females experiencing less than one-third of the incidence observed in males due to differences in risk factors. While the contribution of epidemiological factors remained stable for females after 2020, demographic factors, particularly population aging, continued to influence liver cancer incidence. Unlike males, where demographic and epidemiological factors combined to decrease incidence, the stable contribution of epidemiological factors in females could not offset the impact of demographics. This may result in a potential increase in the absolute number of liver cancer cases in females, albeit at a lower level compared to males. Despite this, the incidence rate and number of liver cancer cases in females in Hong Kong remain significantly lower than in males, underscoring the need for gender-specific risk factor considerations in liver cancer prevention and control strategies.

This study possesses several notable strengths that contribute to understanding liver cancer incidence in Hong Kong. Firstly, it is the most extensive epidemiological study on age-period-cohort associations with liver cancer, analyzing nearly all liver cancer cases in the region over 30 years. This extensive dataset provides robust and reliable estimates of the period and cohort effects on liver cancer incidence. Moreover, the study offers valuable insights into the future incidence of liver cancer in Hong Kong. By projecting the trends observed in the data, the study provides essential information for public health planning and resource allocation.

However, our study also has some limitations. First, liver cancer can be classified into two subtypes: HCC and intrahepatic cholangiocarcinomas, each with distinct risk factors, carcinogenesis, and epidemiological patterns. However, the HKCaR does not differentiate between these subtypes, which limits our ability to analyze and interpret the data accurately. Second, the HKCaR lacks essential individual-level information, such as hepatitis virus infection status, alcohol consumption, obesity, diabetes, or smoking history. This lack of data on crucial risk factors hampers our ability to comprehensively examine the population’s underlying causes of liver cancer. Third, the population structure and size of Hong Kong were derived from the UN World Population Prospects, which may contain significant biases. These biases could affect the accuracy of our findings and projections. Finally, using different modelling approaches introduces variations that could impact the accuracy of our predictions. Care should be taken in interpreting the results and considering the potential uncertainties associated with the modelling techniques employed. Future research should address our study’s limitations by incorporating subtype-specific data, including individual-level risk factor information, and refining population data.

## Conclusion

The period and cohort risks of liver cancer in Hong Kong have declined. The demographic and epidemiological factors contribute to the observed trend. Specifically, it is plausible that the number of liver cancer cases in males will continue to decrease while there may be an increase in the number of cases among females. Further research and epidemiological evaluation of the disease are needed.

### Electronic supplementary material

Below is the link to the electronic supplementary material.

Supplementary Material 1

## Data Availability

The dataset utilized in this study can be accessed from the Hong Kong Cancer Registry repository, available at the following website: https://www3.ha.org.hk/cancereg/allages.asp.
