# Supplementary material for: Trends and future projections of liver cancer incidence in Hong Kong: a population-based study
Source: Arch Public Health. 2023 Oct 3;81:179. doi: 10.1186/s13690-023-01191-3 (PMC10548600; doi:10.1186/s13690-023-01191-3)
Supplement: Supplementary file 1 — Supplementary Material 1 [file 13690_2023_1191_MOESM1_ESM.docx]

**Supplementary Material**

**Table of content**

**Appendix S1. The decomposition method**

**Appendix S2. Projection of future incidence**

**Table S1. Wald Chi-square tests for estimable parameters in the APC model**

**Table S2. Estimated new cases of age-specific liver cancer in Hong Kong males from 1991 to 2030.**

**Table S3. Estimated new cases of age-specific liver cancer in Hong Kong females from 1991 to 2030.**

**Table S4. Contribution of changes in population ageing, population growth, and age-specific incidence rate to the net change of new cases of liver cancer in Hong Kong males from 1992 to 2030, using 1991 as the reference year.**

**Table S5. Contribution of changes in population ageing, population growth, and age-specific incidence rate to the net change of new cases of liver cancer in Hong Kong females from 1992 to 2030, using 1991 as the reference year.**

**Appendix S1. The decomposition method**

The population decomposition algorithm used in this study has been described in detail in the papers by Cheng et al. [1,2]. In brief, the net change in new cases of liver cancer between 1991 and 2020 in Hong Kong was decomposed into the contributions of population growth, population ageing, and age-specific incidence rate.

The age groups were defined using 5-year increments from 20-24 years to 85 plus (with the 85-89 years age group including individuals aged 85 years and older, as recorded as one group in the database. Let *d_ij_*, *n_ij_*, *m_ij_* and *s_ij_* denote the incident cases, population size, age-specific rate of incidence, and population proportion in the *i*^th^ age group of the year *j*, respectively, (*i* = 1, 2, …,12; *j* = 1, 2). Let *D*_1_ and *D*_2_, *N*_1_ and *N*_2_, *P*_1_ and *P*_2_ represent the total incident cases, population size, and crude rate of incidence in 1991 and 2020.

Using *M_p_*, *M_a,_* and *M_m_* to represent the main effects of the changes in population size, age structure, and incidence rate, and *I_pa_*, *I_pm_*, *I_am,_* and *I_pam_* to represent their two-way and three-way interactions, respectively. In the case of 1990 as the reference year, these terms are calculated as follows:

$M_{p}=\sum_{i=1}^{12} {{\left( N_{2}-N_{1} \right)s}_{i1}m}_{i1}$

$M_{a}=\sum_{i=1}^{12} N_{1}\left( s_{i2}-s_{i1} \right)m_{i1}$

$M_{m}=\sum_{i=1}^{12} {N_{1}s}_{i1}\left( m_{i2}-m_{i1} \right)$

$I_{pa}=\sum_{i=1}^{12} \left( N_{2}-N_{1} \right)\left( s_{i2}-s_{i1} \right)m_{i1}$

$I_{pm}=\sum_{i=1}^{12} \left( N_{2}-N_{1} \right)s_{i1}\left( m_{i2}-m_{i1} \right)$

$I_{am}=\sum_{i=1}^{12} N_{1}\left( s_{i2}-s_{i1} \right)\left( m_{i2}-m_{i1} \right)$

$I_{pam}=\sum_{i=1}^{12} \left( N_{2}-N_{1} \right)\left( s_{i2}-s_{i1} \right)\left( m_{i2}-m_{i1} \right)$

Here, a simplification needs to be made, assuming that the interactions are equally distributed, then the contribution of the three factors can be calculated as follows:

$A{=M}_{a}+½I_{am}+½I_{pa}+⅓I_{pam}$

$P{=M}_{p}+½I_{pm}+½I_{pa}+⅓I_{pam}$

$M{=M}_{m}+½I_{pm}+½I_{am}+⅓I_{pam}$

In the decomposition algorithm, the contributions of population ageing, population growth, and age-specific death rate to the net change in new cases of liver cancer are represented by *A*, *P*, and *M*, respectively. The net change represents the total change in new cases of liver cancer between the two time points.

To calculate the percentage contribution of each factor, the contribution is divided by *D_1_* (total incident cases in the reference year) and multiplied by 100. This provides the percentage representation of each factor's contribution to the net change in new cases of liver cancer.

**References**

1. Cheng X, Yang Y, Schwebel DC, Liu Z, Li L, Cheng P et al. Population ageing and mortality during 1990-2017: A global decomposition analysis. PLoS Med 2020;17:e1003138.

2. Cheng X, Tan L, Gao Y, Yang Y, Schwebel DC , Hu G. A new method to attribute differences in total deaths between groups to population size, age structure and age-specific mortality rate. PLoS One 2019;14:e0216613.

**Appendix S2. Projection of future incidence**

The future incident cases of liver cancer in Hong Kong were projected using the Bayesian age-period-cohort analysis with integrated nested Laplace approximations (INLA). This approach allows for separate effects attributed to age, period, and cohort and enables extrapolation to make projections. Unlike the classical approach, the Bayesian approach does not rely on strong parametric assumptions and provides nonarbitrary and sensible projections.

Based on the expectation that adjacent effects in time might be similar, the Bayesian inference in the age-period-cohort model applies the second-order random walk for smoothing priors of age, period, and cohort effects and project posterior mortality rates. According to this model, each point of effect is predicted by linear extrapolation from its two immediate predecessors, plus a random variance from a normal distribution with a mean zero. The INLA is used with this Bayesian age-period-cohort model to approximate the marginal posterior distributions avoiding any mixing and convergence issues introduced by Markov chain Monte Carlo sampling techniques traditionally used in the Bayesian approach. The Bayesian age-period-cohort analysis was conducted by R-package BAPC (version 0.0.34).

We prepared age-specific incident cases of liver cancer (from 1991 to 2020) and Hong Kong population data (from 1991 to 2030), followed by a 10-year (from 2021 to 2030) retrospective projection using the BAPC function in the R package BAPC.

**References**

1. Riebler, A. and L. Held, Projecting the future burden of cancer: Bayesian age-period-cohort analysis with integrated nested Laplace approximations. Biometrical Journal, 2017. 59(3): p. 531-549.

2. Jacobs, D., et al., Assessment of Age, Period, and Birth Cohort Effects and Trends in Merkel Cell Carcinoma Incidence in the United States. Jama Dermatology, 2021. 157(1): p. 59-65.

**Table S1. Wald Chi-square tests for estimable parameters in the APC model**

| Null Hypothesis | Male | | Female | |
| --- | --- | --- | --- | --- |
|  | Chi-squre | P-value | Chi-squre | P-value |
| NetDrift = 0 | 553.7 | <0.001 | 95.0 | <0.001 |
| All Age Deviations = 0 | 2143.8 | <0.001 | 334.5 | <0.001 |
| All Period RR = 1 | 561.0 | <0.001 | 112.1 | <0.001 |
| All Cohort RR = 1 | 1589.5 | <0.001 | 605.4 | <0.001 |
| All Local Drifts = Net Drift | 252.5 | <0.001 | 139.0 | <0.001 |

**Table S2. Estimated new cases of age-specific liver cancer in Hong Kong males from 1991 to 2030.** The data from 2021 to 2030 were projected.

| Year |  | Number of age-specific liver cases | | | | | | | | | | | | | |
| --- | --- | --- | --- | --- | --- | --- | --- | --- | --- | --- | --- | --- | --- | --- | --- |
|  | 20-24 | 25-29 | 30-34 | 35-39 | 40-44 | 45-49 | 50-54 | 55-59 | 60-64 | 65-69 | 70-74 | 75-79 | 80-84 | 85+ | Total |
| 1991 | 1 | 15 | 28 | 52 | 88 | 71 | 119 | 149 | 200 | 140 | 138 | 87 | 23 | 13 | 1124 |
| 1992 | 5 | 12 | 31 | 61 | 86 | 80 | 118 | 154 | 199 | 181 | 143 | 69 | 50 | 19 | 1208 |
| 1993 | 6 | 11 | 24 | 65 | 86 | 99 | 108 | 153 | 215 | 201 | 138 | 86 | 53 | 19 | 1264 |
| 1994 | 5 | 11 | 23 | 53 | 85 | 116 | 124 | 185 | 204 | 190 | 172 | 87 | 45 | 20 | 1320 |
| 1995 | 5 | 5 | 16 | 56 | 93 | 99 | 105 | 139 | 198 | 185 | 109 | 96 | 49 | 22 | 1177 |
| 1996 | 4 | 8 | 27 | 49 | 80 | 126 | 145 | 165 | 202 | 185 | 159 | 91 | 57 | 32 | 1330 |
| 1997 | 5 | 7 | 17 | 71 | 72 | 119 | 123 | 139 | 203 | 193 | 167 | 89 | 55 | 23 | 1283 |
| 1998 | 1 | 10 | 16 | 40 | 88 | 118 | 124 | 162 | 197 | 209 | 151 | 106 | 57 | 16 | 1295 |
| 1999 | 5 | 5 | 8 | 38 | 85 | 132 | 117 | 138 | 173 | 198 | 144 | 99 | 44 | 28 | 1214 |
| 2000 | 1 | 13 | 12 | 53 | 88 | 119 | 147 | 126 | 170 | 184 | 132 | 103 | 37 | 32 | 1217 |
| 2001 | 2 | 5 | 11 | 25 | 87 | 133 | 155 | 139 | 163 | 217 | 181 | 103 | 57 | 31 | 1309 |
| 2002 | 2 | 5 | 9 | 29 | 85 | 116 | 137 | 140 | 141 | 175 | 171 | 122 | 57 | 40 | 1229 |
| 2003 | 1 | 4 | 10 | 24 | 71 | 123 | 157 | 150 | 151 | 167 | 169 | 127 | 56 | 48 | 1258 |
| 2004 | 1 | 4 | 14 | 23 | 79 | 116 | 152 | 136 | 150 | 183 | 173 | 121 | 84 | 40 | 1276 |
| 2005 | 2 | 5 | 8 | 29 | 66 | 129 | 142 | 181 | 153 | 187 | 170 | 130 | 84 | 36 | 1322 |
| 2006 | 1 | 3 | 13 | 21 | 50 | 127 | 178 | 180 | 152 | 161 | 187 | 127 | 73 | 52 | 1325 |
| 2007 | 1 | 6 | 7 | 24 | 45 | 111 | 143 | 184 | 183 | 167 | 164 | 134 | 89 | 44 | 1302 |
| 2008 | 1 | 6 | 18 | 25 | 40 | 112 | 178 | 187 | 159 | 167 | 176 | 123 | 79 | 47 | 1318 |
| 2009 | 1 | 1 | 8 | 31 | 46 | 101 | 197 | 199 | 198 | 160 | 163 | 142 | 84 | 52 | 1383 |
| 2010 | 2 | 2 | 12 | 24 | 38 | 90 | 184 | 226 | 211 | 151 | 154 | 158 | 88 | 58 | 1398 |
| 2011 | 0 | 6 | 7 | 24 | 48 | 74 | 173 | 213 | 234 | 167 | 139 | 140 | 100 | 68 | 1393 |
| 2012 | 0 | 4 | 10 | 20 | 35 | 63 | 157 | 204 | 230 | 174 | 158 | 151 | 98 | 58 | 1362 |
| 2013 | 4 | 2 | 6 | 22 | 30 | 72 | 153 | 233 | 240 | 190 | 153 | 131 | 99 | 66 | 1401 |
| 2014 | 2 | 5 | 8 | 10 | 44 | 58 | 140 | 223 | 223 | 192 | 149 | 135 | 110 | 67 | 1366 |
| 2015 | 0 | 3 | 3 | 12 | 33 | 58 | 103 | 228 | 232 | 188 | 165 | 139 | 130 | 61 | 1355 |
| 2016 | 1 | 4 | 5 | 22 | 32 | 48 | 127 | 227 | 277 | 221 | 148 | 125 | 84 | 69 | 1390 |
| 2017 | 0 | 1 | 12 | 19 | 39 | 53 | 124 | 195 | 246 | 262 | 148 | 136 | 93 | 77 | 1405 |
| 2018 | 0 | 1 | 2 | 16 | 29 | 58 | 101 | 194 | 234 | 258 | 168 | 123 | 94 | 80 | 1358 |
| 2019 | 0 | 1 | 4 | 16 | 39 | 48 | 83 | 200 | 263 | 239 | 232 | 134 | 97 | 91 | 1447 |
| 2020 | 1 | 1 | 1 | 12 | 12 | 49 | 69 | 155 | 242 | 217 | 177 | 121 | 107 | 94 | 1258 |
| 2021 | 0 | 1 | 4 | 11 | 24 | 47 | 75 | 143 | 243 | 245 | 204 | 124 | 98 | 93 | 1312 |
| 2022 | 0 | 1 | 3 | 10 | 23 | 45 | 72 | 128 | 229 | 249 | 208 | 132 | 92 | 95 | 1287 |
| 2023 | 0 | 1 | 3 | 9 | 21 | 42 | 70 | 115 | 213 | 252 | 211 | 142 | 86 | 96 | 1261 |
| 2024 | 0 | 1 | 3 | 8 | 20 | 39 | 68 | 105 | 196 | 253 | 213 | 152 | 84 | 96 | 1238 |
| 2025 | 0 | 1 | 3 | 7 | 18 | 37 | 66 | 97 | 178 | 249 | 217 | 160 | 86 | 95 | 1214 |
| 2026 | 0 | 1 | 2 | 7 | 17 | 34 | 63 | 92 | 160 | 240 | 221 | 166 | 90 | 96 | 1189 |
| 2027 | 0 | 1 | 2 | 6 | 15 | 32 | 60 | 89 | 144 | 227 | 225 | 170 | 97 | 96 | 1164 |
| 2028 | 0 | 1 | 2 | 6 | 14 | 30 | 56 | 86 | 130 | 212 | 228 | 173 | 105 | 95 | 1138 |
| 2029 | 0 | 0 | 2 | 5 | 13 | 28 | 53 | 84 | 119 | 195 | 230 | 176 | 112 | 94 | 1111 |
| 2030 | 0 | 0 | 1 | 5 | 12 | 26 | 50 | 82 | 110 | 178 | 227 | 180 | 118 | 94 | 1083 |

**Table S3. Estimated new cases of age-specific liver cancer in Hong Kong females from 1991 to 2030.** The data from 2021 to 2030 were projected

| Year |  | Number of age-specific liver cases | | | | | | | | | | | | | |
| --- | --- | --- | --- | --- | --- | --- | --- | --- | --- | --- | --- | --- | --- | --- | --- |
|  | 20-24 | 25-29 | 30-34 | 35-39 | 40-44 | 45-49 | 50-54 | 55-59 | 60-64 | 65-69 | 70-74 | 75-79 | 80-84 | 85+ | Total |
| 1991 | 1 | 3 | 4 | 6 | 19 | 12 | 15 | 36 | 36 | 40 | 48 | 37 | 33 | 22 | 312 |
| 1992 | 3 | 4 | 10 | 11 | 12 | 10 | 11 | 36 | 45 | 56 | 60 | 55 | 21 | 26 | 360 |
| 1993 | 0 | 6 | 3 | 11 | 8 | 12 | 19 | 31 | 56 | 51 | 47 | 48 | 34 | 35 | 361 |
| 1994 | 0 | 6 | 6 | 9 | 17 | 13 | 13 | 43 | 46 | 54 | 66 | 49 | 41 | 16 | 379 |
| 1995 | 4 | 5 | 5 | 9 | 17 | 20 | 21 | 37 | 51 | 61 | 52 | 57 | 35 | 25 | 399 |
| 1996 | 0 | 7 | 10 | 10 | 15 | 17 | 17 | 32 | 52 | 58 | 55 | 49 | 45 | 27 | 394 |
| 1997 | 3 | 2 | 5 | 9 | 16 | 20 | 20 | 33 | 51 | 59 | 72 | 52 | 31 | 33 | 406 |
| 1998 | 0 | 2 | 6 | 11 | 23 | 21 | 20 | 21 | 33 | 65 | 67 | 35 | 46 | 29 | 379 |
| 1999 | 0 | 3 | 3 | 5 | 13 | 24 | 18 | 16 | 35 | 56 | 59 | 55 | 25 | 39 | 351 |
| 2000 | 3 | 1 | 5 | 6 | 12 | 19 | 27 | 20 | 39 | 47 | 66 | 47 | 34 | 35 | 361 |
| 2001 | 0 | 1 | 4 | 11 | 10 | 15 | 17 | 21 | 33 | 48 | 55 | 48 | 29 | 31 | 323 |
| 2002 | 1 | 2 | 1 | 5 | 8 | 12 | 18 | 24 | 25 | 49 | 60 | 55 | 32 | 49 | 341 |
| 2003 | 1 | 1 | 3 | 4 | 16 | 18 | 26 | 26 | 30 | 55 | 69 | 51 | 40 | 48 | 388 |
| 2004 | 0 | 0 | 1 | 10 | 7 | 17 | 23 | 20 | 31 | 60 | 66 | 55 | 50 | 44 | 384 |
| 2005 | 0 | 1 | 1 | 6 | 13 | 16 | 24 | 38 | 32 | 61 | 53 | 74 | 54 | 52 | 425 |
| 2006 | 0 | 1 | 3 | 6 | 11 | 13 | 16 | 39 | 41 | 51 | 58 | 75 | 49 | 50 | 413 |
| 2007 | 0 | 2 | 0 | 5 | 6 | 13 | 19 | 30 | 33 | 50 | 79 | 46 | 62 | 40 | 385 |
| 2008 | 0 | 4 | 3 | 7 | 10 | 20 | 39 | 48 | 28 | 42 | 52 | 73 | 55 | 44 | 425 |
| 2009 | 0 | 4 | 2 | 8 | 6 | 22 | 30 | 39 | 40 | 36 | 68 | 79 | 55 | 56 | 445 |
| 2010 | 2 | 2 | 5 | 7 | 7 | 15 | 28 | 40 | 57 | 48 | 63 | 68 | 65 | 54 | 461 |
| 2011 | 0 | 1 | 7 | 4 | 10 | 10 | 27 | 35 | 50 | 45 | 65 | 77 | 60 | 65 | 456 |
| 2012 | 0 | 1 | 3 | 11 | 10 | 16 | 22 | 30 | 40 | 43 | 55 | 63 | 66 | 65 | 425 |
| 2013 | 0 | 1 | 3 | 7 | 8 | 11 | 24 | 38 | 53 | 77 | 46 | 55 | 66 | 54 | 443 |
| 2014 | 0 | 2 | 0 | 2 | 13 | 11 | 24 | 47 | 48 | 61 | 56 | 74 | 59 | 80 | 477 |
| 2015 | 1 | 0 | 3 | 4 | 6 | 10 | 16 | 32 | 49 | 58 | 61 | 63 | 66 | 64 | 433 |
| 2016 | 0 | 0 | 1 | 2 | 6 | 6 | 21 | 46 | 45 | 64 | 48 | 58 | 51 | 69 | 417 |
| 2017 | 0 | 1 | 2 | 5 | 9 | 11 | 21 | 36 | 43 | 58 | 43 | 52 | 70 | 72 | 423 |
| 2018 | 0 | 2 | 1 | 5 | 7 | 12 | 18 | 26 | 35 | 56 | 58 | 44 | 53 | 64 | 381 |
| 2019 | 0 | 0 | 1 | 3 | 3 | 6 | 19 | 34 | 56 | 64 | 67 | 43 | 55 | 76 | 427 |
| 2020 | 0 | 0 | 4 | 1 | 5 | 14 | 17 | 33 | 45 | 70 | 65 | 49 | 79 | 90 | 472 |
| 2021 | 0 | 1 | 1 | 3 | 6 | 9 | 16 | 31 | 49 | 66 | 75 | 53 | 59 | 92 | 461 |
| 2022 | 0 | 1 | 1 | 3 | 5 | 9 | 16 | 31 | 50 | 68 | 78 | 60 | 55 | 97 | 474 |
| 2023 | 0 | 1 | 1 | 3 | 5 | 9 | 16 | 30 | 51 | 70 | 82 | 70 | 52 | 103 | 493 |
| 2024 | 0 | 1 | 1 | 3 | 5 | 9 | 16 | 29 | 51 | 73 | 85 | 80 | 51 | 108 | 512 |
| 2025 | 0 | 0 | 1 | 3 | 5 | 9 | 16 | 28 | 51 | 75 | 89 | 88 | 55 | 111 | 531 |
| 2026 | 0 | 0 | 1 | 2 | 5 | 9 | 16 | 28 | 52 | 78 | 94 | 96 | 61 | 115 | 557 |
| 2027 | 0 | 0 | 1 | 2 | 5 | 9 | 16 | 29 | 51 | 81 | 99 | 102 | 72 | 117 | 584 |
| 2028 | 0 | 0 | 1 | 2 | 5 | 10 | 17 | 30 | 51 | 85 | 105 | 110 | 85 | 119 | 620 |
| 2029 | 0 | 0 | 1 | 2 | 5 | 10 | 17 | 30 | 51 | 88 | 112 | 118 | 100 | 125 | 659 |
| 2030 | 0 | 0 | 1 | 2 | 5 | 10 | 18 | 32 | 53 | 92 | 120 | 128 | 114 | 135 | 710 |

**Table S4. Contribution of changes in population ageing, population growth, and age-specific incidence rate to the net change of new cases of liver cancer in Hong Kong males from 1992 to 2030, using 1991 as the reference year.**

| year | Population aging(%) | Population growth(%) | Epidemiological change(%) | Net change(%) |
| --- | --- | --- | --- | --- |
| 1992 | 15 (1.3) | 20 (1.7) | 50 (4.4) | 84 (7.5) |
| 1993 | 32 (2.8) | 39 (3.5) | 69 (6.1) | 140 (12.5) |
| 1994 | 48 (4.3) | 60 (5.4) | 87 (7.8) | 196 (17.4) |
| 1995 | 66 (5.9) | 78 (6.9) | -91 (-8.1) | 53 (4.7) |
| 1996 | 88 (7.8) | 102 (9.1) | 16 (1.4) | 206 (18.3) |
| 1997 | 107 (9.5) | 122 (10.8) | -70 (-6.2) | 159 (14.1) |
| 1998 | 120 (10.7) | 145 (12.9) | -94 (-8.4) | 171 (15.2) |
| 1999 | 142 (12.6) | 159 (14.2) | -211 (-18.8) | 90 (8.0) |
| 2000 | 159 (14.2) | 174 (15.5) | -241 (-21.4) | 93 (8.3) |
| 2001 | 193 (17.1) | 195 (17.4) | -203 (-18) | 185 (16.5) |
| 2002 | 209 (18.6) | 199 (17.7) | -303 (-27) | 105 (9.3) |
| 2003 | 232 (20.6) | 208 (18.5) | -305 (-27.2) | 134 (11.9) |
| 2004 | 251 (22.3) | 215 (19.1) | -313 (-27.9) | 152 (13.5) |
| 2005 | 275 (24.5) | 224 (19.9) | -301 (-26.8) | 198 (17.6) |
| 2006 | 310 (27.5) | 233 (20.8) | -342 (-30.4) | 201 (17.9) |
| 2007 | 326 (29.0) | 241 (21.5) | -389 (-34.6) | 178 (15.8) |
| 2008 | 353 (31.4) | 252 (22.4) | -411 (-36.5) | 194 (17.3) |
| 2009 | 392 (34.8) | 267 (23.7) | -399 (-35.5) | 259 (23.0) |
| 2010 | 422 (37.5) | 278 (24.7) | -425 (-37.9) | 274 (24.4) |
| 2011 | 439 (39.1) | 287 (25.5) | -457 (-40.7) | 269 (23.9) |
| 2012 | 454 (40.4) | 296 (26.3) | -512 (-45.5) | 238 (21.2) |
| 2013 | 480 (42.7) | 311 (27.7) | -514 (-45.7) | 277 (24.6) |
| 2014 | 494 (44.0) | 318 (28.3) | -570 (-50.8) | 242 (21.5) |
| 2015 | 519 (46.1) | 325 (29.0) | -613 (-54.6) | 231 (20.6) |
| 2016 | 537 (47.8) | 342 (30.5) | -613 (-54.6) | 266 (23.7) |
| 2017 | 562 (50.0) | 354 (31.5) | -635 (-56.5) | 281 (25.0) |
| 2018 | 586 (52.1) | 356 (31.7) | -708 (-63.0) | 234 (20.8) |
| 2019 | 626 (55.7) | 372 (33.1) | -675 (-60.1) | 323 (28.7) |
| 2020 | 630 (56.1) | 356 (31.6) | -852 (-75.8) | 134 (11.9) |
| 2021 | 660 (58.7) | 365 (32.5) | -837 (-74.5) | 188 (16.7) |
| 2022 | 683 (60.7) | 365 (32.5) | -885 (-78.7) | 163 (14.5) |
| 2023 | 705 (62.7) | 365 (32.5) | -933 (-83.0) | 137 (12.2) |
| 2024 | 726 (64.6) | 365 (32.5) | -977 (-86.9) | 114 (10.1) |
| 2025 | 744 (66.2) | 366 (32.6) | -1020 (-90.8) | 90 (8.0) |
| 2026 | 756 (67.2) | 369 (32.8) | -1059 (-94.3) | 65 (5.8) |
| 2027 | 764 (68.0) | 372 (33.1) | -1096 (-97.5) | 40 (3.6) |
| 2028 | 769 (68.4) | 376 (33.5) | -1132 (-100.7) | 14 (1.2) |
| 2029 | 774 (68.9) | 380 (33.8) | -1168 (-103.9) | -13 (-1.2) |
| 2030 | 779 (69.3) | 383 (34.1) | -1203 (-107.1) | -41 (-3.6) |

**Table S4. Contribution of changes in population ageing, population growth, and age-specific incidence rate to the net change of new cases of liver cancer in Hong Kong females from 1992 to 2030, using 1991 as the reference year.**

| year | Population aging(%) | Population growth(%) | Epidemiological change(%) | Net change(%) |
| --- | --- | --- | --- | --- |
| 1992 | 2 (0.6) | 8 (2.5) | 38 (12.3) | 48 (15.4) |
| 1993 | 3 (1.1) | 15 (4.9) | 30 (9.7) | 49 (15.7) |
| 1994 | 5 (1.6) | 24 (7.7) | 38 (12.2) | 67 (21.5) |
| 1995 | 8 (2.6) | 34 (10.8) | 45 (14.5) | 87 (27.9) |
| 1996 | 10 (3.1) | 41 (13.3) | 31 (9.9) | 82 (26.3) |
| 1997 | 12 (3.8) | 52 (16.5) | 31 (9.8) | 94 (30.1) |
| 1998 | 16 (5.1) | 59 (19.0) | -8 (-2.7) | 67 (21.5) |
| 1999 | 18 (5.8) | 66 (21.2) | -45 (-14.5) | 39 (12.5) |
| 2000 | 18 (5.7) | 75 (24.1) | -44 (-14.1) | 49 (15.7) |
| 2001 | 21 (6.6) | 79 (25.2) | -88 (-28.3) | 11 (3.5) |
| 2002 | 29 (9.3) | 86 (27.7) | -87 (-27.8) | 29 (9.3) |
| 2003 | 37 (11.8) | 97 (31.2) | -58 (-18.7) | 76 (24.4) |
| 2004 | 41 (13.1) | 103 (32.9) | -71 (-22.9) | 72 (23.1) |
| 2005 | 51 (16.3) | 113 (36.3) | -51 (-16.4) | 113 (36.2) |
| 2006 | 52 (16.6) | 119 (38.1) | -69 (-22.3) | 101 (32.4) |
| 2007 | 55 (17.5) | 122 (39.0) | -103 (-33.1) | 73 (23.4) |
| 2008 | 69 (22.1) | 133 (42.6) | -89 (-28.5) | 113 (36.2) |
| 2009 | 76 (24.4) | 142 (45.6) | -85 (-27.4) | 133 (42.6) |
| 2010 | 77 (24.7) | 152 (48.7) | -80 (-25.7) | 149 (47.8) |
| 2011 | 85 (27.3) | 157 (50.2) | -98 (-31.4) | 144 (46.2) |
| 2012 | 91 (29.0) | 157 (50.4) | -135 (-43.2) | 113 (36.2) |
| 2013 | 94 (30.1) | 167 (53.4) | -129 (-41.5) | 131 (42.0) |
| 2014 | 109 (35.0) | 177 (56.7) | -121 (-38.8) | 165 (52.9) |
| 2015 | 107 (34.4) | 175 (56.2) | -162 (-51.9) | 121 (38.8) |
| 2016 | 120 (38.4) | 177 (56.9) | -192 (-61.6) | 105 (33.7) |
| 2017 | 130 (41.7) | 183 (58.8) | -203 (-64.9) | 111 (35.6) |
| 2018 | 130 (41.8) | 181 (58.1) | -242 (-77.7) | 69 (22.1) |
| 2019 | 149 (47.8) | 193 (61.8) | -227 (-72.8) | 115 (36.9) |
| 2020 | 167 (53.4) | 205 (65.7) | -211 (-67.7) | 160 (51.3) |
| 2021 | 175 (56.0) | 206 (66.0) | -232 (-74.3) | 149 (47.8) |
| 2022 | 187 (60.1) | 211 (67.7) | -237 (-75.9) | 162 (51.9) |
| 2023 | 201 (64.6) | 218 (69.8) | -238 (-76.4) | 181 (58.0) |
| 2024 | 216 (69.3) | 224 (71.8) | -240 (-77.0) | 200 (64.1) |
| 2025 | 232 (74.5) | 230 (73.9) | -244 (-78.2) | 219 (70.2) |
| 2026 | 250 (80.2) | 238 (76.4) | -244 (-78.1) | 245 (78.5) |
| 2027 | 268 (86.0) | 246 (78.9) | -242 (-77.7) | 272 (87.2) |
| 2028 | 289 (92.5) | 256 (82) | -236 (-75.7) | 308 (98.7) |
| 2029 | 310 (99.5) | 266 (85.1) | -229 (-73.4) | 347 (111.2) |
| 2030 | 336 (107.6) | 278 (89.0) | -215 (-69.0) | 398 (127.6) |
